# Salicine

**Published:** 1874-10

**Authors:** N. D. Tobey

**Affiliations:** West Columbia, West Virginia


### SALICINE.

BY N. D. TOBEY, M.D., WEST COLUMBIA, WEST VIRGINIA.

Salicine is the active or medicinal principle of willow bark. It is derived principally from the salix helix, while the bark of the other varieties of willow possess it, as well as the different varieties of poplar. It is found more abundantly, however, in the bark of the salix helix, consequently that variety is said to be almost the only one used in its manufacture. As the process of manufacture falls entirely within the provincc of the manufacturing chemist, and not within the sphere of the therapeutist, we will not notice that process in this paper. Salicine is a white powder, somewhat resembling sulph. quinia, but distinguishable by being heavier, less delicately crystallized, and not so adherent in flakes. It is said, however, to be used in adulterating quinia, and I believe I have seen specimens of quinia which contained it. It is not soluble in sulph. acid like quinine, but turns to a red color when the acid is applied to it, thereby making the acid the test for its presence in a suspected article of quinia. It is soluble in hot water, and moderately so in cold water. Its taste is intensely bitter, but does not remain so long in the mouth as the taste of quinia. The tonic properties of willow bark were known long before the discovery of salicine, and the bark was extensively used upon the European continent (in many portions of which the tree is indigenous as well as on this continent) in domestic practice as well as by the profession. It was used in infusion or decoction, and also in powder. The bark was taken off of the tree, and when dried, became quilled. It was used as a simple tonic as well as an antiperiodic. As a tonic, the testimony was strongly in its favor, but not very strong as an antiperiodic, consequently it gradually fell into disuse, until the discovery of its active principle, called salicine. Salicine was procured in its pure state by Leraux, an apothecary of Vitry, France, in the year 1828. It had been obtained by several German and Italian chemists in an impure state, prior to that year (*Dunglison's New Remedies*, seventh edition, p. 641). The well known therapeutic properities of the bark of the willow soon caused salicine to be extensively used, and especially in France.

Miquel, a French physician, was among the first to institute experiments, and regarded from his observations that it was as efficient a remedy in intermittent fever as quinia, but requiring larger doses.

Many other observers soon verified the opinion of Miquel, and declared in favor of the antiperiodic virtues of the drug, and classed it as next to quinia for efficiency in intermittent and remittent fevers. But while in Europe, and especially in France, salicine was extensively used and favorably regarded, it does not appear that in our country it was much used, and seems to have met with little favor. About the year 1845, Dr. Lawson, then Surgeon-General of the United States army, authorized experiments to be instituted with quinia and salicine, in order that the relative value of each might be made known as antispasmodics. Dr. E. D. Fenner, of New Orleans, having experimented fully with the drugs, gave it as his opinion that salicine was fully as efficacious as quinia, but required to be given in doses three times as large. But it appears that Dr. Fenner's opinion was based more upon the relative price of the drugs than upon their actual relative power or therapeutic agency. The price of quinia at that time was no higher than that of salicine, and his conclusions were that seventy-five cents' worth of salicine was no more efficacious in twenty cases of intermittent fever than twenty-five cents' worth of quinine.

The therapeutic properties of salicine can be regarded as tonic and antiperiodic, and testimony is not wanting to class it as such an agent. Without giving further the opinions of authors upon the drug, we will now endeavor to show that salicine is a drug of no little utility. Our first acquaintance with it was during the years of 1864 and 1865, when the price of quinia was very high on account of the war. We did not have, however, much experience with it as an antiperiodic, from the fact that the country in which we were practicing, was very little affected with malaria. We used it as a simple tonic in cases where, in our judgment, quinine was indicated, and saw no reason to doubt its utility as a therapeutic agent. Since then we have been using it, and while we are free to confess that we do not regard it as certain in its action in intermittent or remittent diseases as quinine, yet, in many instances, it can safely be relied on, and on account of its being less irritating to the stomach, can, in some cases, be used more advantageously in some forms of neuralgia—those which simulate migraine, or sick headache, we have found it beneficial. The first case in which we gave it a fair trial, was one known popularly as sun-pain. The patient had suffered with a pain above his left eye, periodically, for years, the character of which was to remit at about 12 o'clock in the day, and then come on violently about 6 o'clock, or sunrise, the next morning. I used hypodermic injections of morphia and sulph. quinia, and relieved him of his first attack, in which I saw him in about ten days. In about six months it again made its appearance, and was relieved after about ten days of suffering by the same treatment. The pain again appeared, after a lapse of about six months, and the same treatment was again tried: morphia, hypodermically, and quinine, for about two days, with no relief whatever. I then prescribed about ten grains of salicine at a dose, to be repeated in two hours, beginning about 3 o'clock in the morning, and had the satisfaction of seeing my patient relieved after using the prescription two mornings.

In this case, the morphia injections and quinine in large doses always severely nauseated the stomach. The salicine did not nauseate at all, and, besides, did not produce the ordinary condition known as cinchonism. The second case was not of the same character, but the patient complained of a dull, throbbing headache, commencing about daylight, and leaving him about noon. It had annoyed him for several days, but he was busily engaged at his work, and never ceased on account of the pain. I gave him six powders of salicine, containing about six grains each, ordering him to take one at bed-time, and two early in the morning, at an interval of about two hours. The six powders gave him entire relief.

Others, of a like character, might be enumerated here, but the above will suffice to illustrate the action of the drug in cases of a neuralgic form, which seem to depend upon what might be called centric causes, as no appreciable cause for the pain could be found in arrest of secretion on initiation of internal viscera. In such cases, we believe it to be as powerfully antiperiodic as quinine.

Now, to what extent salicine may be antiperiodic in regular intermittent fever, and what influence it may have over malaria in the blood, we will not pretend to say. Our use of it has not been sufficient to aid us in arriving at anything like a fixed conclusion on this point. If quinine exerts its antiperiodic process as a spanæmic, we are inclined to think that salicine does also; while in small doses it certainly acts as a mild tonic, aiding digestion and assimilation. Upon this ground, we have administered it in cases of *chronic diarrhœa, with benefit,* combining it with rhubarb, in small doses, and repeating it every four hours. As a simple tonic, its therapeutic process evidently depends upon its bitter principle and power to enrich the blood, while it evidently has further action upon the nervous system. When given in large dóses, it does not produce in many instances that peculiar condition which quinia does, commonly called cinchonism; while if it does in others, only to a very limited extent.

While we do not regard salicine as efficacious or valuable an agent as quinine, we nevertheless regard it as a very valuable addition to our materia medica and to the practitioner, who supplies his own drugs, and in many instances gives both his labor and drugs gratis. It would be a means of saving, as it is only about one-third the price of quinine. We would not, however, urge such a reason as an apology for its use, if we did not have confidence in its virtues as a therapeutic agent. We employ it in powder, combined with an equal amount of white sugar, or in pill, or dissolved in hot water. As it is perfectly soluble in alcohol, it could be employed as a tincture. No substance as a simple salt or chemical product, is more free from impurities than salicine, and this demonstrates the very valuable aid of organic chemistry to the therapeutist in freeing many vegetable preparations from all inert principles by extracting the active, thereby lessening the dose and making medicines more agreeable to the patient. Upon the authority of Von Dem Bush, a German physician, salicine can be employed in cases where there is a heavy secretion from mucous membranes. He instances bronchitis and leucorrhæa, in which cases he used it in decoction with polygola amara, or Iceland moss. We have never as yet employed it in such cases, but believe it to possess alterative powers over mucous surfaces, as instanced in the fact that it will aid digestion and assimilation, where there is atony of the digestive organs. From what we have said, from our own use of the drug and the testimony of others, we feel justified in drawing the following conclusions;

1. Salicine is a safe and reliable tonic and antiperiodic, second in favor to quinia.

2. It can be used in cases where there is gastric irritation to better advantage than quinia.

3. It is an alterative to mucous surfaces, and can be safely employed as such.

4. It deserves to be more freely used by the profession, as a matter of economy to both physician and patient.

